# Supplementary material for: Nanoribbons self-assembled from short peptides demonstrate the formation of polar zippers between β-sheets
Source: Nat Commun. 2018 Nov 30;9:5118. doi: 10.1038/s41467-018-07583-2 (PMC6269506; doi:10.1038/s41467-018-07583-2)
Supplement: Supplementary file 1 — Supplementary Information [file 41467_2018_7583_MOESM1_ESM.pdf]

Nanoribbons self-assembled from short peptides demonstrate the formation of polar zippers between β-sheets

Wang et al.

Supplementary Information

Nanoribbons self-assembled from short peptides demonstrate the formation of polar zippers between β-sheets

Meng Wang[1], Jiqian Wang[1]*, Peng Zhou[1], Jing Deng[2], Yurong Zhao[1], Yawei Sun[1], Wei Yang[1], Dong Wang[1], Zongyi Li[3], Xuzhi Hu[3], Stephen M. King[4], Sarah E. Rogers[4], Henry Cox[3], Thomas A. Waigh[3], Jun Yang[2], Jian R. Lu[3]* and Hai Xu[1]*

[1] Centre for Bioengineering and Biotechnology, College of Chemical Engineering, China University of Petroleum (East China), 66 Changjiang West Road, Qingdao 266580, China

[2] National Center for Magnetic Resonance in Wuhan, Key Laboratory of Magnetic Resonance in Biological Systems, State Key Laboratory of Magnetic Resonance and Atomic and Molecular Physics, Wuhan Institute of Physics and Mathematics, Chinese Academy of Sciences, Wuhan 430071, China

[3] Biological Physics Group, School of Physics and Astronomy, The University of Manchester, Manchester M13 9PL, United Kingdom

[4] ISIS Pulsed Neutron Source, STFC Rutherford Appleton Laboratory, Didcot, Oxon OX11 0QX, United Kingdom

E-mails: jqwang@upc.edu.cn (J.W.); j.lu@manchester.ac.uk (J.R.L.); xuh@upc.edu.cn (H.X.)

Supplementary Figures

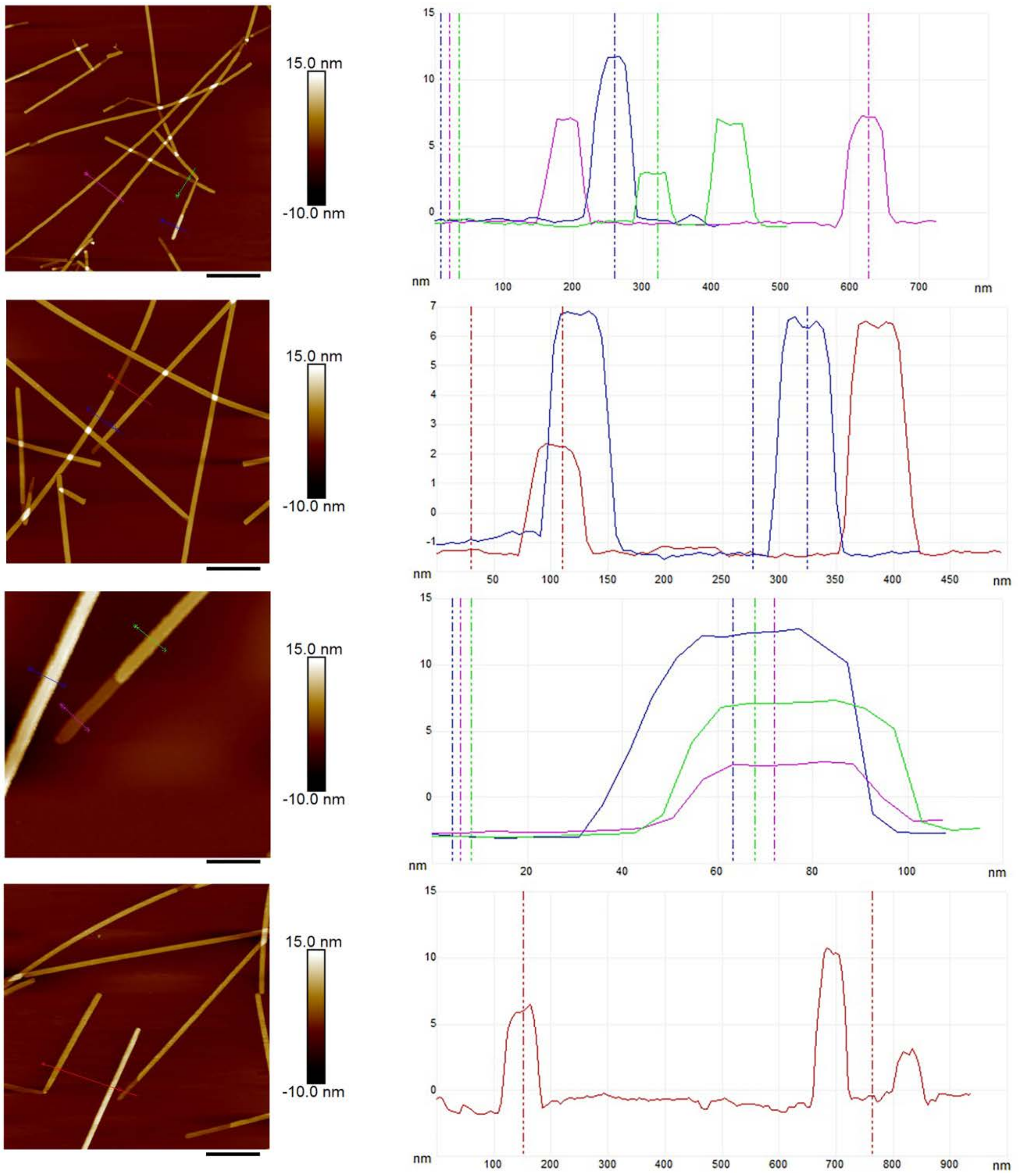

Supplementary Fig. 1 Representative AFM height images and sectional height profiles. These results suggest that the Ac-I_3QGK-NH_2 nanoribbons are multilayered and each layer is composed of an interdigitated peptide bilayer. The bilayer thickness was found to be mainly between 3.5 and 4.0 nm based on many AFM sectional height profiles (~30 individual nanoribbons). Here, we just show 4 representative ones. Scale bar represents 800, 500, 160, and 500 nm, respectively, from the top to the bottom.

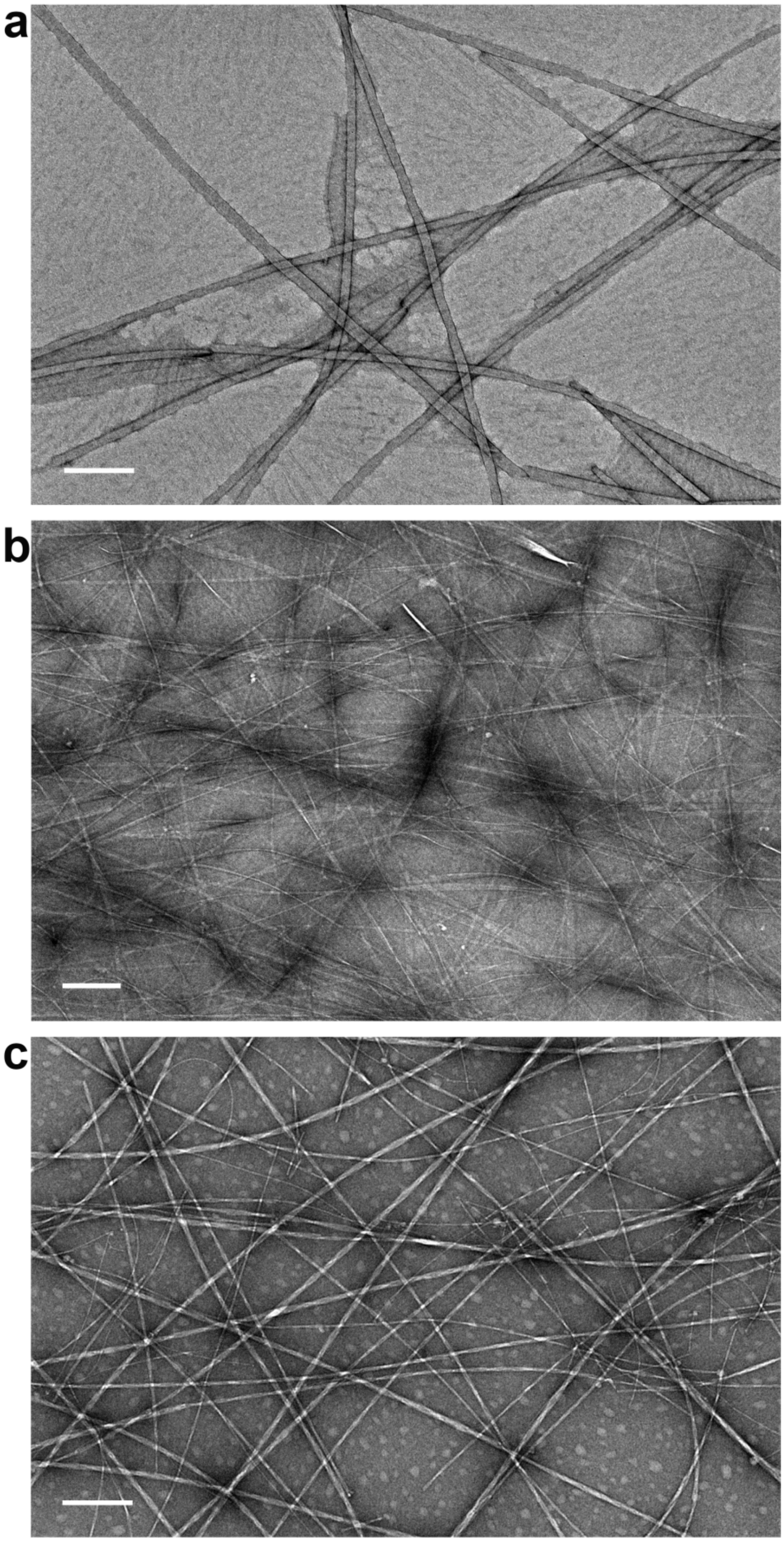

Supplementary Fig. 2 TEM images of diluted peptide assemblies. **a** Ac-I_3QGK-NH_2 (2 mM). **b** Ac-I_3SGK-NH_2 (2 mM). **c** Ac-I_3GGK-NH_2 (4 mM). Scale bar, 200 nm. The samples were obtained by 4-fold dilution from 8 or 16 mM peptide solutions.

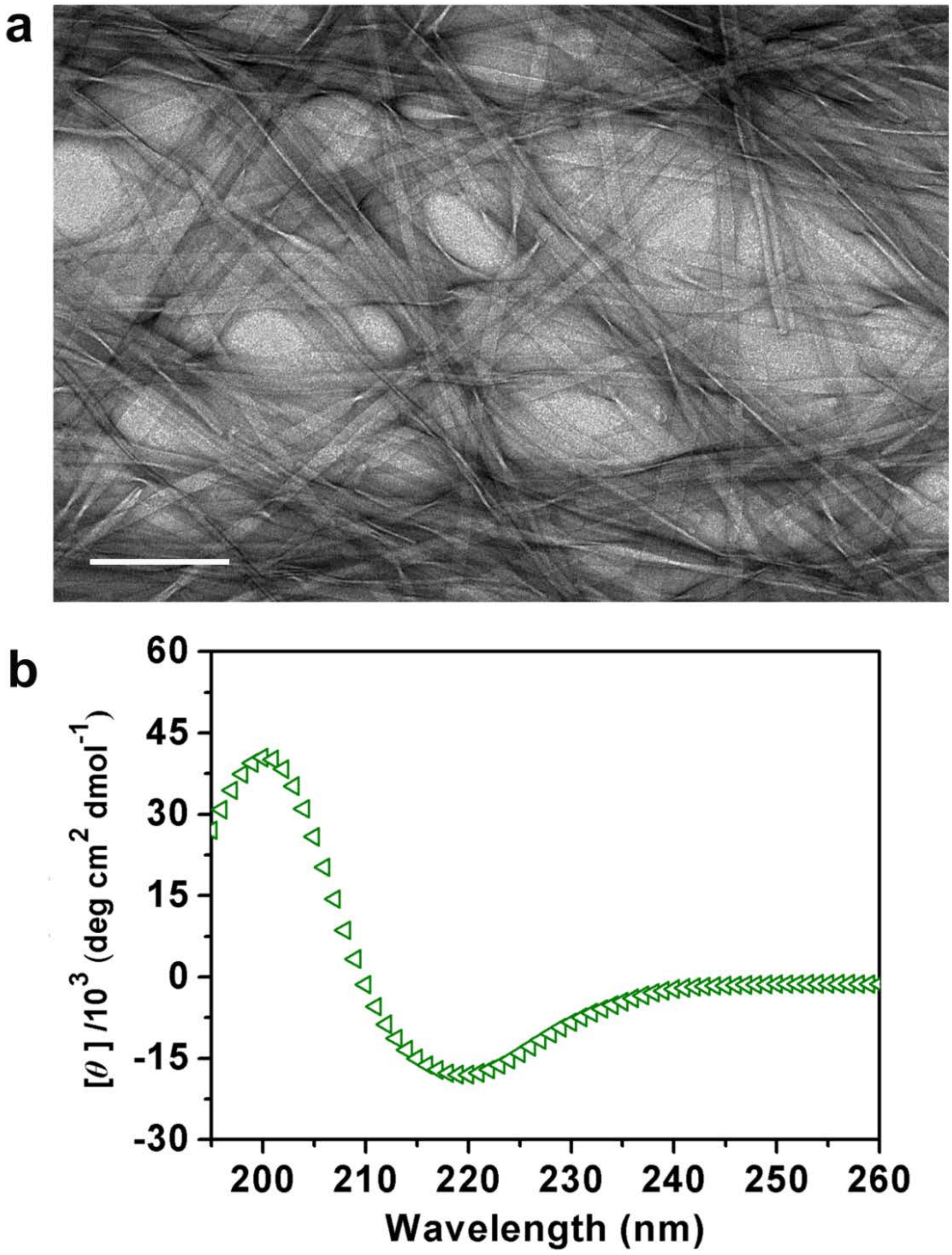

Supplementary Fig. 3 TEM image and CD spectrum of 8 mM Ac-I_3NGK-NH_2. **a** TEM image. Scale bar, 200 nm. **b** CD spectrum (molar ellipticity [θ] as a function of wavelength). The peptide solution was incubated at pH 7.0 for one week.

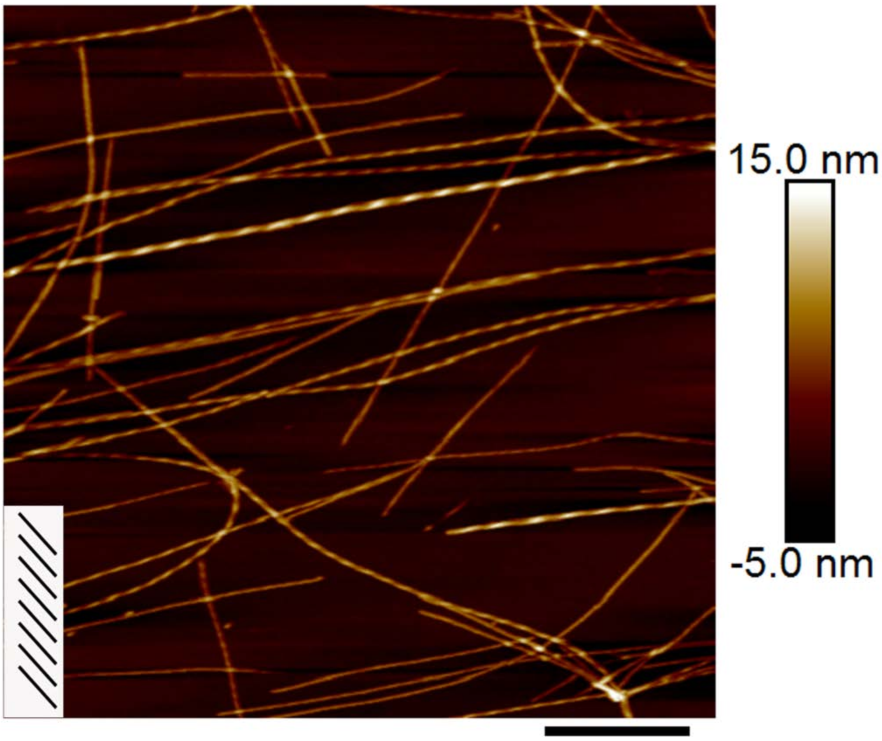

Supplementary Fig. 4 Tapping-mode height AFM image of Ac-I_3GGK-NH_2. Scale bar represents 500 nm, and the nanofibers are left-hand twisted. The peptide nanofibers were formed by 16 mM Ac-I_3GGK-NH_2 at pH 7.0 after incubation for 1 week.

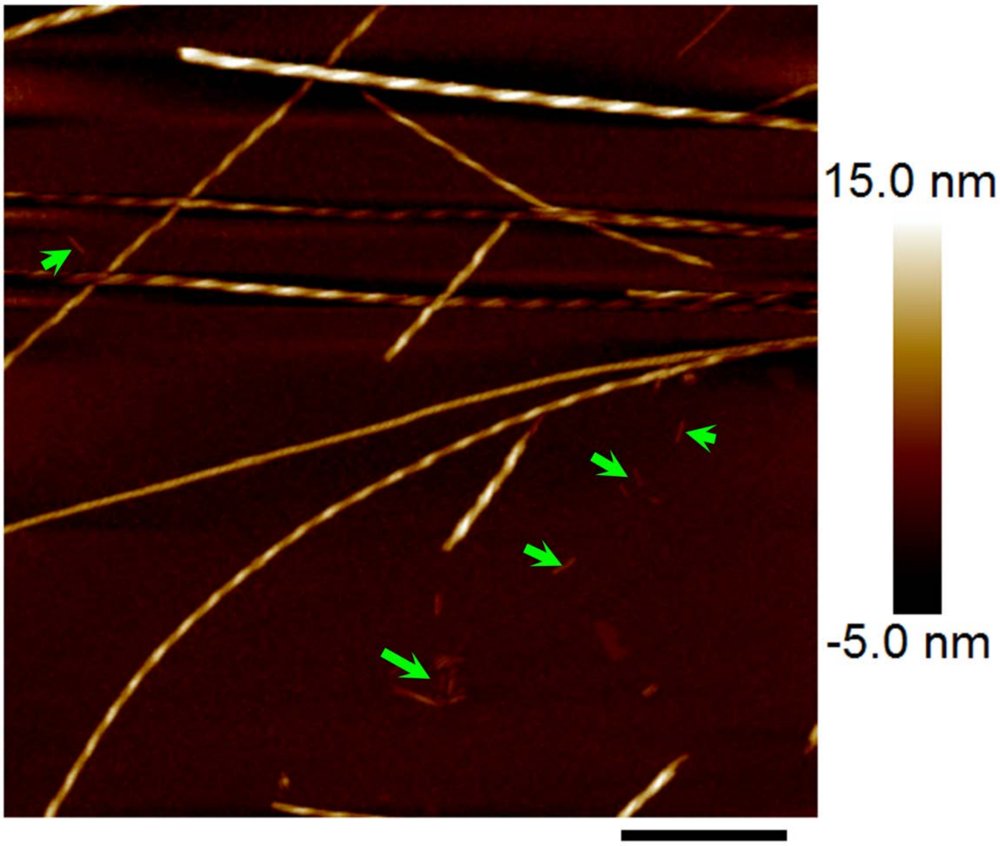

Supplementary Fig. 5 AFM image of 16 mM Ac-I_3GGK-NH_2. In addition to long fibers, shorter and thinner protofibrils (indicated by green arrows) were also observed. The peptide solution was incubated at pH 7.0 for 1 week. Scale bar, 500 nm.

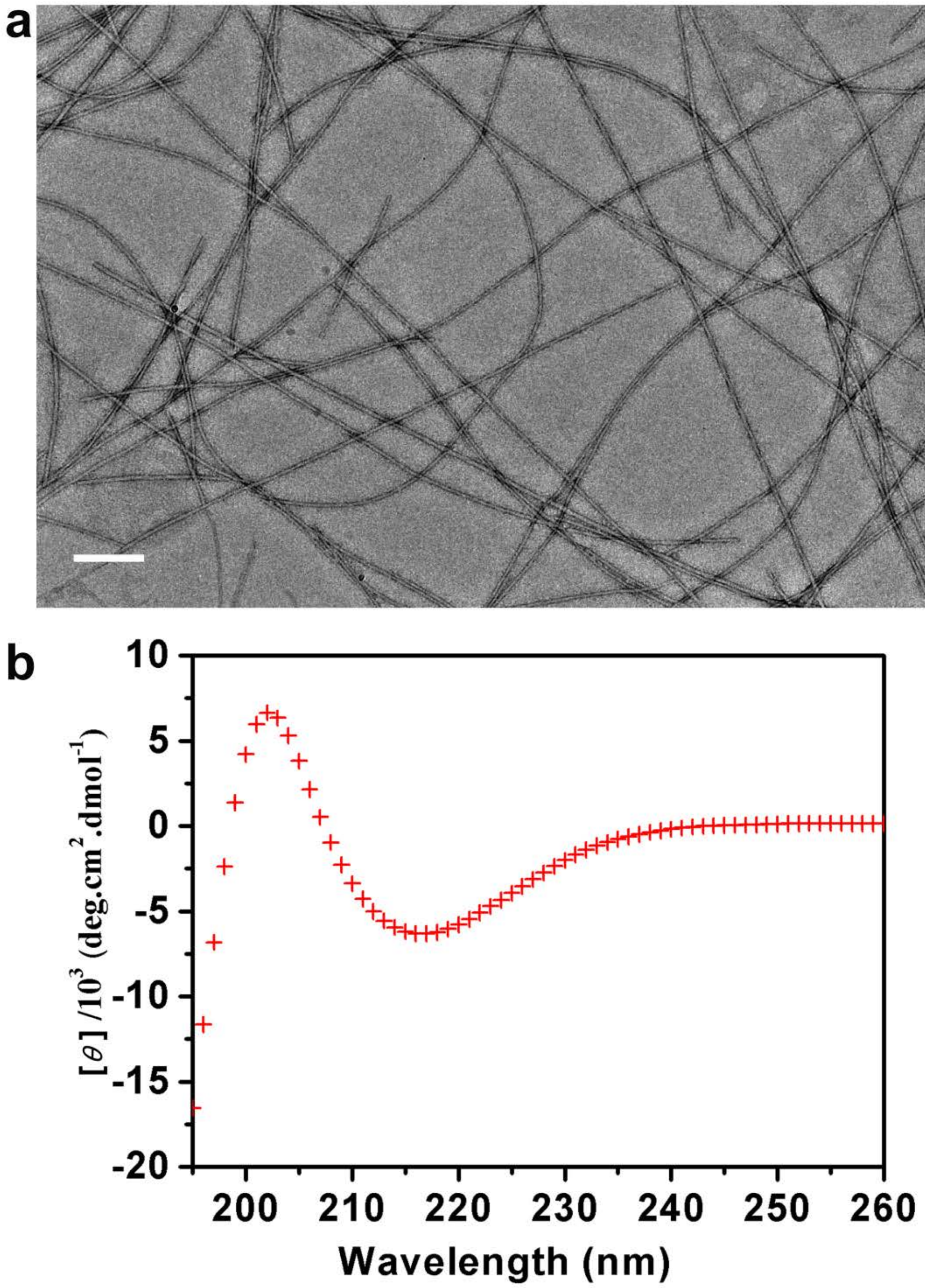

Supplementary Fig. 6 TEM image and CD spectrum of 8 mM Ac-I_3LGK-NH_2. **a** TEM image. Scale bar, 200 nm. **b** CD spectrum. The peptide solution was incubated at pH 7.0 for one week.

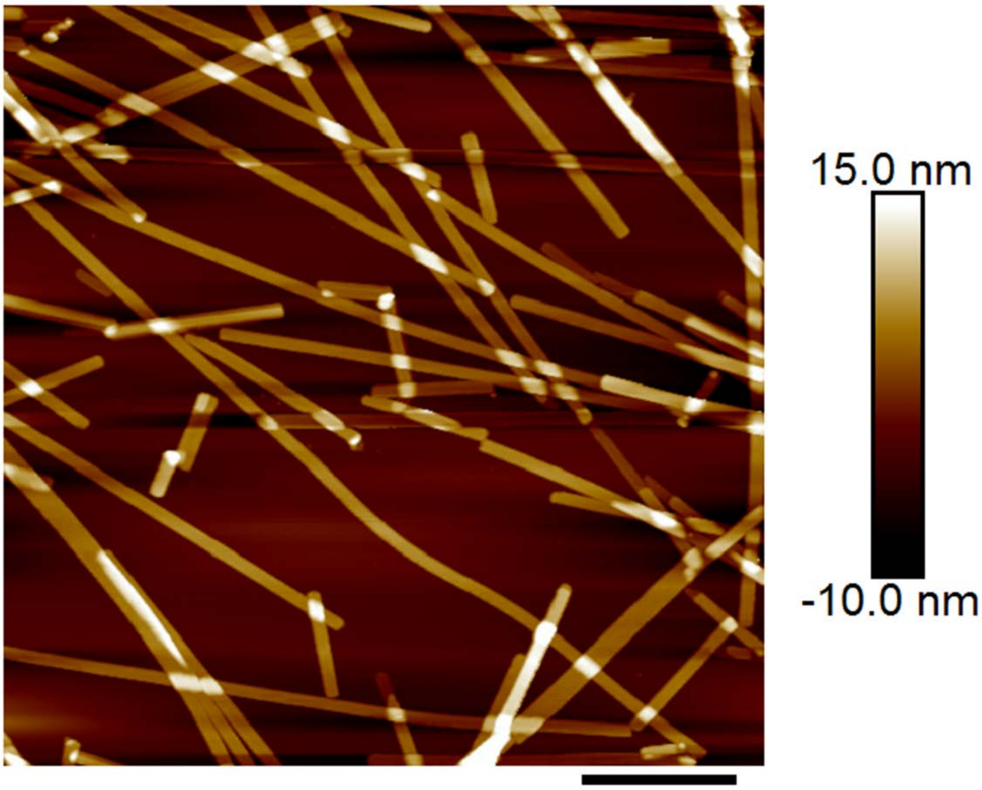

Supplementary Fig. 7 AFM height image of Ac-I_3QGK-NH_2 nanoribbons. The sample was incubated for three months. Scale bar, 510 nm.

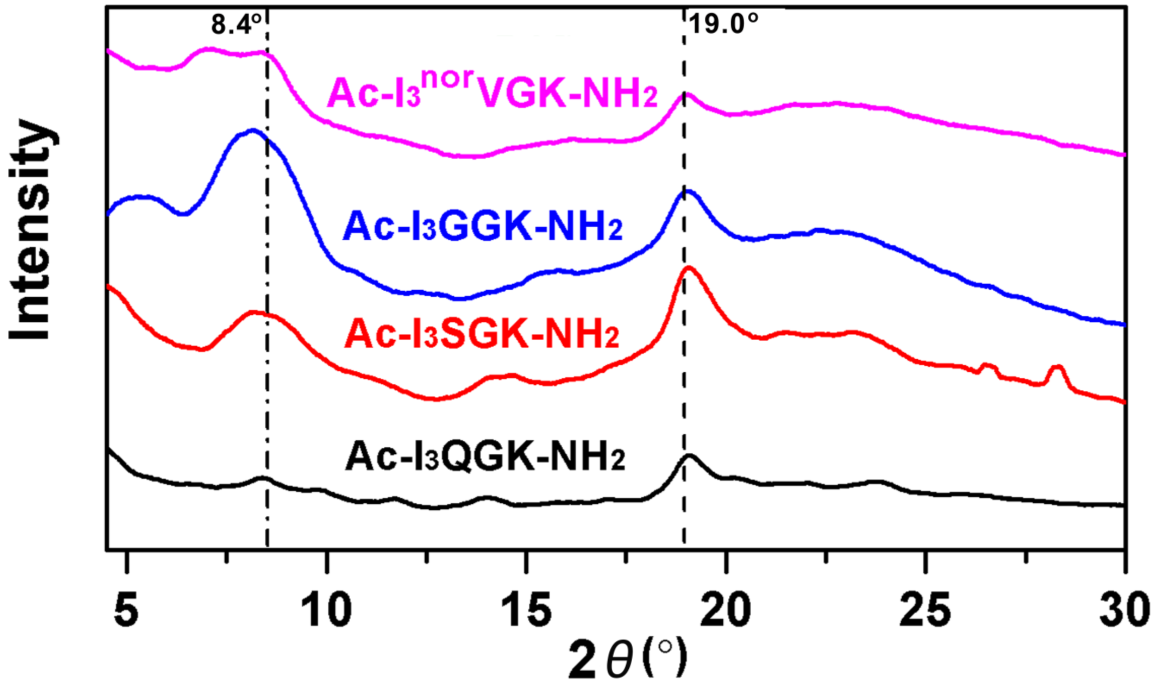

Supplementary Fig. 8 Powder XRD patterns of the designed peptides. After incubation for 1 week, the peptide solutions were lyophilized for 2 days to get powders for XRD measurements.

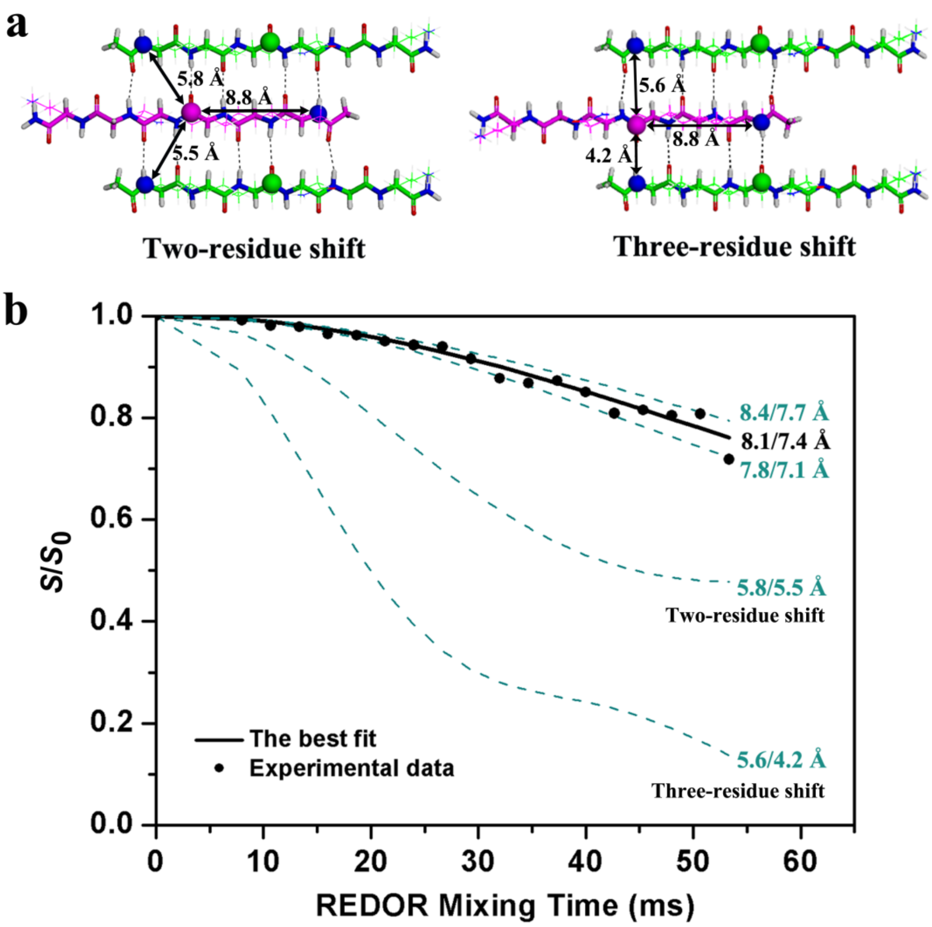

Supplementary Fig. 9 Molecular packing modes. **a** Anti-parallel trimers that were the central core of an Ac-I_3QGK-NH_2 oligomer of 6 strands × 4 sheets during MD simulations, with one- or two-residue shift, respectively. The simulated intra- and inter-molecular ^{13}C-^{15}N distances (^{13}C: pink or green sphere; ^{15}N: blue sphere) are also indicated. **b** Experimental $^{13}C\{^{15}N\}$ REDOR data (black dots) of [^{15}N]Ile1[1-^{13}C]Ile3-Ac-I_3QGK-NH_2 nanoribbons and the best fit (black solid line). The dashed lines are the calculated REDOR curves with the indicated inter-strand ^{13}C-^{15}N distances.

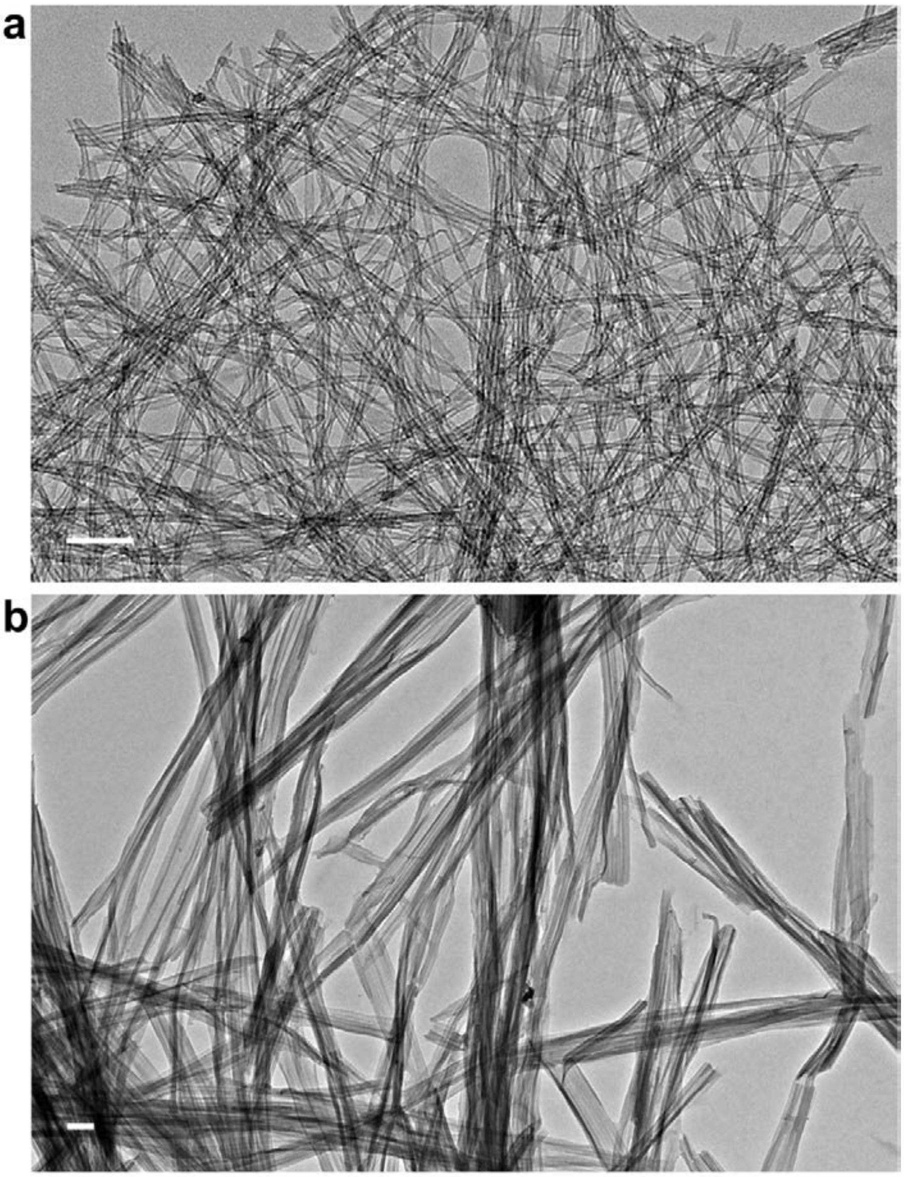

Supplementary Fig. 10 Silica nanostructures templated by peptide self-assemblies. **a** Ac-I_3^{nor}VGK-NH_2 nanofibers. **b** Ac-I_3QGK-NH_2 nanoribbons. Scale bar, 100 nm. These silica nanostructures were obtained through the sol-gel reaction of tetraethoxysilane (TEOS) templated by the peptide nanostructures. In a typical experiment, 40 μL of TEOS was dissolved in 2 mL ethanol, followed by their immediate mixing with 2 mL of 8 mM Ac-I_3^{nor}VGK-NH_2 or Ac-I_3QGK-NH_2 solution (pH 7.0). After reaction for 1 week at room temperature, the silica/peptide composite was collected by ultracentrifugation. The transparent gel-like precipitate collected was copiously rinsed with ethanol and water, and then lyophilized for TEM characterizations.

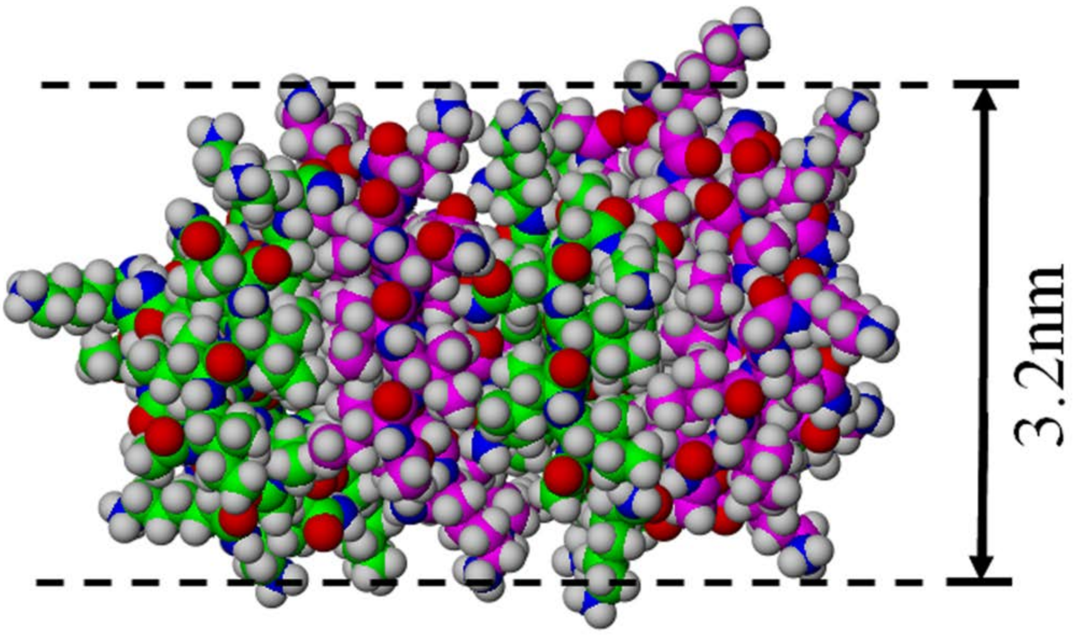

Supplementary Fig. 11 The simulated height of the Ac-I_3QGK-NH_2 oligomer of 6 strands × 4 sheets with one-residue shift.

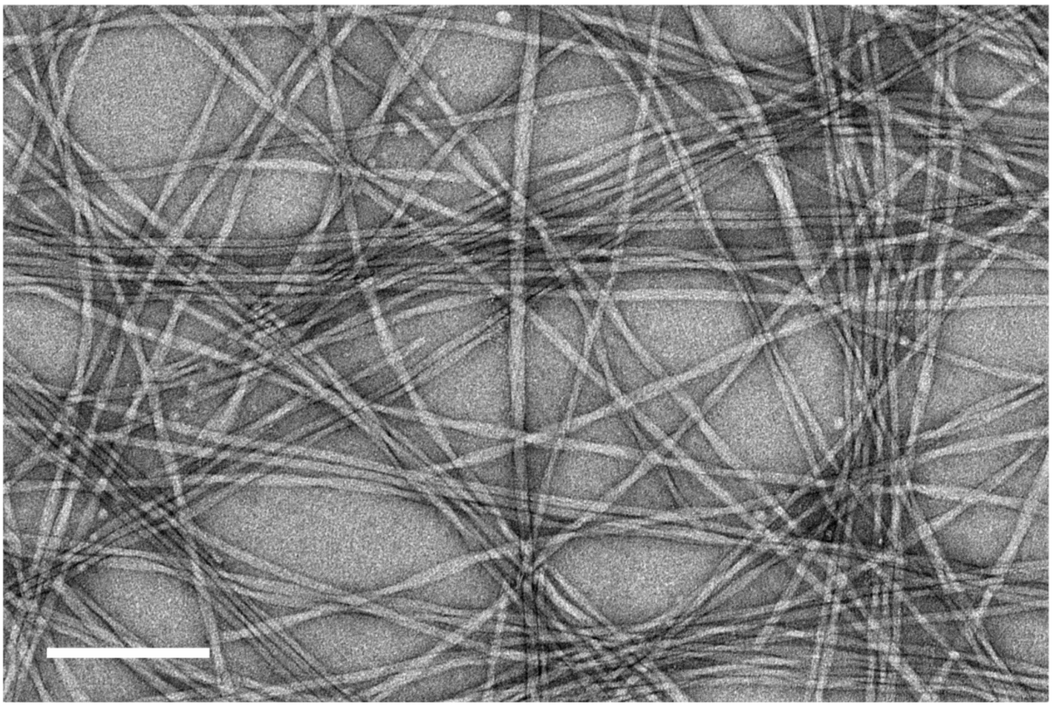

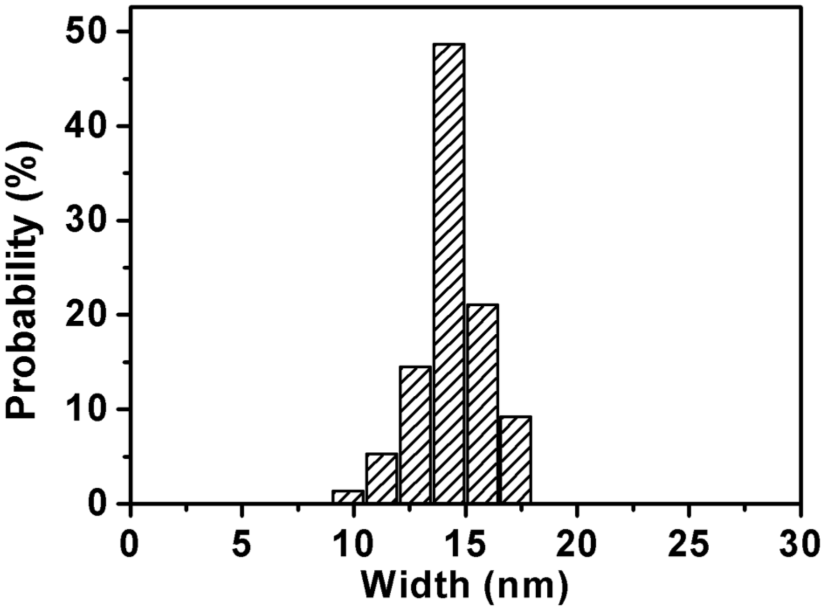

Supplementary Fig. 12 TEM image of 8 mM Ac-I_2QIGK-NH_2 nanofibers and their width distribution. Scale bar, 200 nm. The sample was incubated at pH 7.0 for 1 week, and the width distribution histogram was based on measurements of ~100 individual fibers.

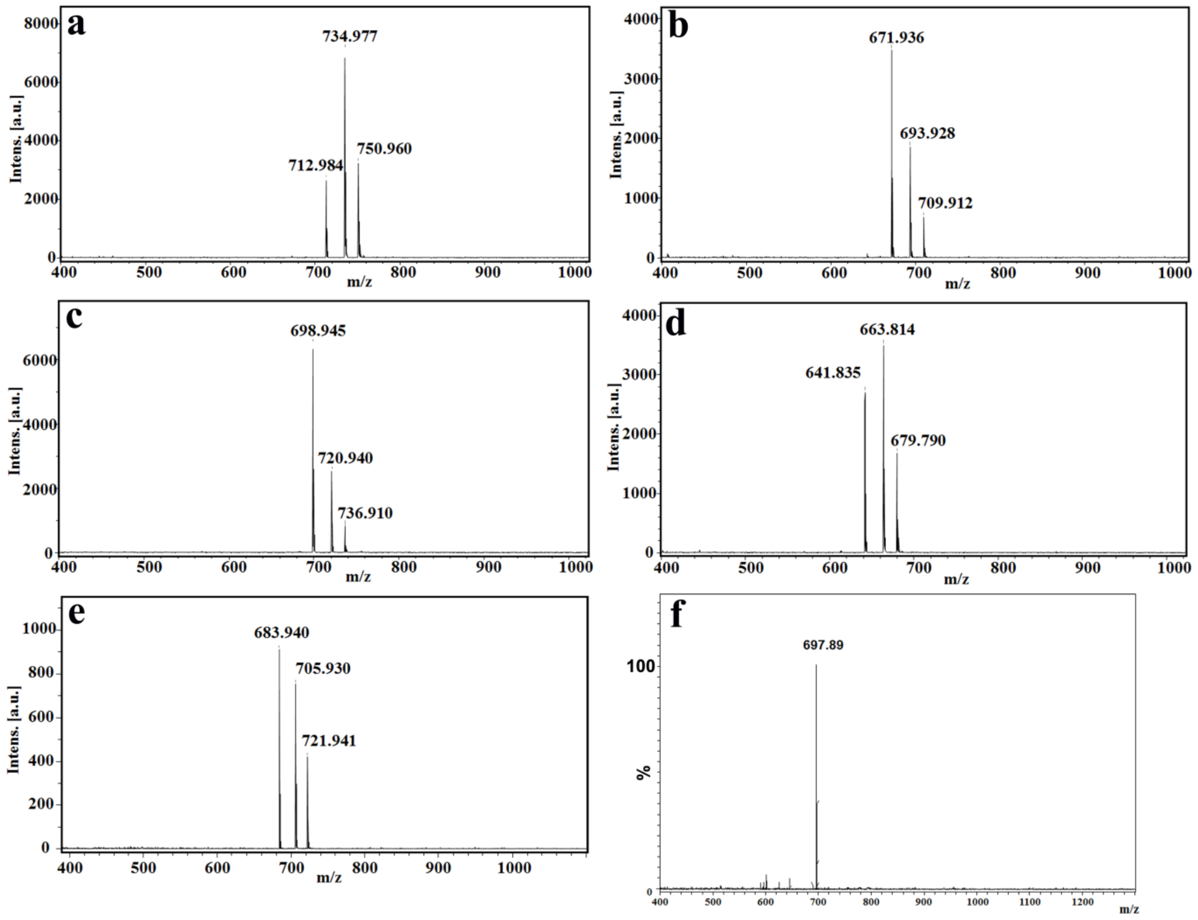

Supplementary Fig. 13 MALDI-TOF and ESI mass spectra. **a** Ac-I_3QGK-NH_2. **b** Ac-I_3SGK-NH_2. **c** Ac-I_3NGK-NH_2. **d** Ac-I_3GGK-NH_2. **e** Ac-I_3^{nor}VGK-NH_2. **f** Ac-I_3LGK-NH_2. The observed molecular ion peaks, corresponding to the H^+, Na^+, and K^+ adducts, indicate the correct sequences.

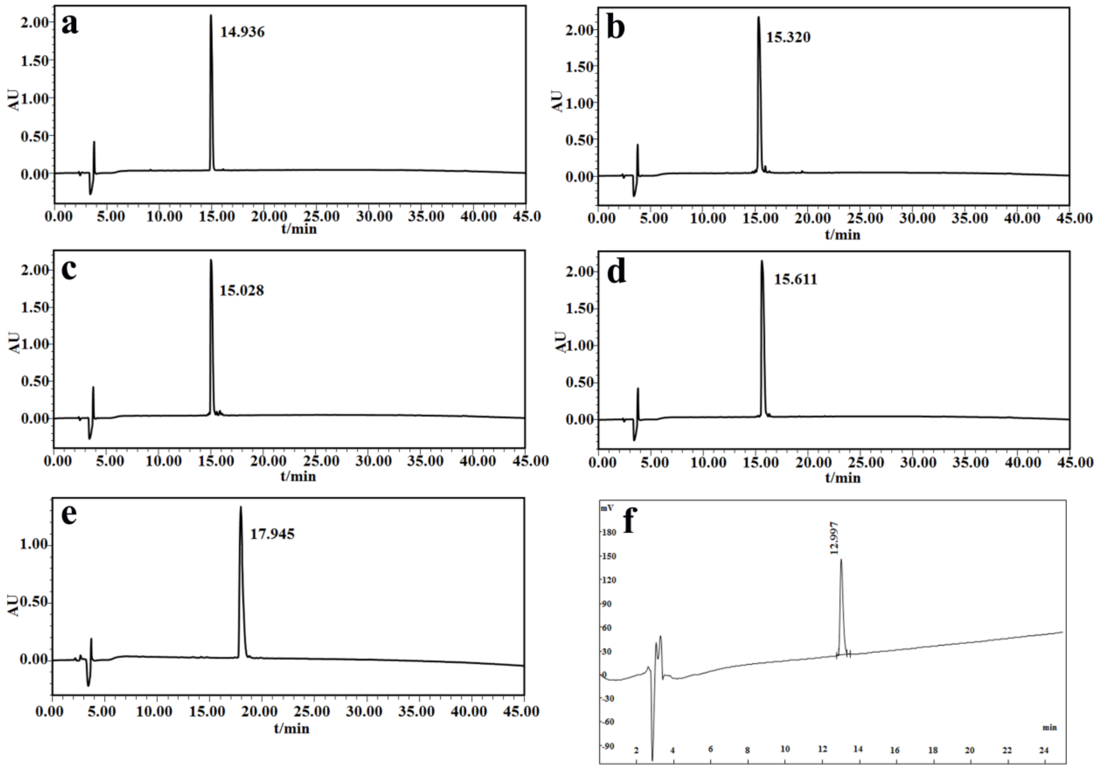

Supplementary Fig. 14 HPLC profiles. **a** Ac-I_3QGK-NH_2. **b** Ac-I_3SGK-NH_2. **c** Ac-I_3NGK-NH_2. **d** Ac-I_3GGK-NH_2. **e** Ac-I_3^{nor}VGK-NH_2. **f** Ac-I_3LGK-NH_2. All these profiles indicate high purity (>98%). Note that the HPLC measurements for the first five peptides were performed with the same HPLC conditions while the HPLC condition for the last sample (Ac-I_3LGK-NH_2) was different, with respect to the used columns and eluents.

Supplementary Tables

Supplementary Table 1 The fitting models applied to, and the optimal structural parameters extracted from, the SANS data from Ac-I_3XGK-NH_2 (X=Q, S, and N) as shown in Fig. 3 of the main manuscript. The fitting was performed in the absence of size polydispersity, giving rise to larger χ^2/N_{pts} values.

Peptide & Concentration	Ac-I_3QGK-NH_2			Ac-I_3SGK-NH_2		Ac-I_3NGK-NH_2
	8 mM	8 mM	2 mM	8 mM	2 mM	8 mM
Fitting model	ECM [a]	ECM+ ECM	ECM	ECM	ECM	ECM
p1_Effective Volume Fraction (%)	0.70	0.69	0.09	0.56	0.09	0.85
p1_Background	0.010	0.010	0.007	0.010	0.003	0.010
p1_Minor Radius (Å)	42	43	42	16.5	15.4	16
p1_Axial Ratio	4.8	5	4.8	7	6.7	11.5
p1_Length (Å)	>1000	>1000	>1000	>1000	>1000	>1000
p1_Sld_Cyl ($\times 10^{-6}$Å^{-2}) [b]	4.0	4.0	4.0	4.0	4.0	4.0
p1_Sld_Sol ($\times 10^{-6}$Å^{-2}) [b]	6.35	6.35	6.35	6.35	6.35	6.35
p2_Effective Volume Fraction (%)	/	0.76	/	/	/	/
p2_Background	/	0	/	/	/	/
p2_Minor Radius (Å)	/	12	/	/	/	/
p2_Axial Ratio	/	1.0	/	/	/	/
p2_Length (Å)	/	25	/	/	/	/
p2_Sld_Cyl ($\times 10^{-6}$Å^{-2})	/	5.0	/	/	/	/
p2_Sld_Sol ($\times 10^{-6}$Å^{-2})	/	6.35	/	/	/	/
Scale Factor	/	1	/	/	/	/
χ^2/N_{pts}	98.8	53.1	5.7	24.2	4.2	8.8

[a] ECM denotes the elliptical cylinder model. [b] Sld_Cyl and SLD_Sol are the scattering length density (ρ) of scattering entities (cylinders) and the solvent (D_2O), respectively.

Supplementary Table 2 The fitting models applied to, and the optimal structural parameters extracted from, the SANS data from Ac-I_3XGK-NH_2 (X=G, ^{nor}V, and L) as shown in Fig. 3 of the main manuscript. The fitting was performed in the presence of size polydispersity for the radius and Kuhn length of the long and thick nanofibers.

Peptides & Concentrations	Ac-I_3GGK-NH_2			Ac-I_3^{nor}VGK-NH_2	Ac-I_3LGK-NH_2
	16 mM	16 mM	4 mM	8 mM	8 mM
Fitting model	FCM [a]	FCM+ FCM	FCM+ FCM	FCM	FCM
p1_Effective Volume Fraction (%)	0.42	0.24	0.06	0.84	0.85
p1_Background	0.013	0.013	0.004	0.009	0.042
p1_Length (Å)	>1000	>1000	>1000	>1000	>500
p1_Kuhn_Length (Å)	62	35	153	498	282
σ/<Kuhn length> [b]	0.41	0.31	0.12	0.48	0.14
p1_Radius (Å)	48	52	63	39.3	44
σ/<radius>	0.21	0.08	0.07	0.20	0.11
p1_Sld_Cyl ($\times10^{-6}$Å^{-2}) [c]	4.0	4.0	4.5	4.0	4.0
p1_Sld_Sol ($\times10^{-6}$Å^{-2}) [c]	6.35	6.35	6.35	6.35	6.35
p2_Effective Volume Fraction (%)	/	1.5	0.41	/	/
p2_Background	/	0	0	/	/
p2_Length (Å)	/	359	210	/	/
p2_Kuhn_Length (Å)	/	90	53	/	/
p2_Radius (Å)	/	12	7	/	/
p2_Sld_Cyl ($\times10^{-6}$Å^{-2})	/	5.0	5.0	/	/
p2_Sld_Sol ($\times10^{-6}$Å^{-2})	/	6.35	6.35	/	/
Scale Factor	/	1	1	/	/
χ^2/N_{pts} [d]	208	10.6	1.6	8.5	2.9

[a] FCM denotes the flexible cylinder model. [b] σ = standard deviation of the lognormal distribution and < > = mean value. [c] Sld_Cyl and SLD_Sol are the scattering length density (ρ) of scattering entities (cylinders) and the solvent (D_2O), respectively. [d] χ^2/N_{pts} values were significantly decreased in the presence of size polydispersity for the radius and Kuhn length of the long nanofibers, in comparison with those assuming an absence of size polydispersity.
